# AI-Driven Career Guidance to Reduce Vocational Students’ Career Path Anxiety through Skills Mapping, Adaptive Mentoring, and Labor Market Intelligence

**DOI:** 10.12688/f1000research.174858.1

**Published:** 2026-02-19

**Authors:** Retyana Wahrini, Hasbi Hasbi, Muhammad Nuruzzaman, Nuzulul Alifin Nur, Pramudita Budiastuti, Magy Gaspersz, Andry Tanggu Mara

**Affiliations:** 1 Vocational and Technology Education, Universitas Negeri Yogyakarta Program Pascasarjana, Yogyakarta, Special Region of Yogyakarta, Indonesia; 2 Departement of Electronic Engineering, Universitas Negeri Makassar, Makassar, South Sulawesi, Indonesia; 3 Civil Engineering Applied Bachelor's Degree Program (D-IV), Universitas Negeri Yogyakarta, Yogyakarta, Special Region of Yogyakarta, Indonesia; 4 Civil Engineering and Planning Education, Universitas Negeri Yogyakarta, Yogyakarta, Special Region of Yogyakarta, Indonesia; 5 Vocational Education in Electronics Engineering, Universitas Ahmad Dahlan, Yogyakarta, Special Region of Yogyakarta, Indonesia; 6 Educational Research and Evaluation, Universitas Negeri Yogyakarta, Yogyakarta, Special Region of Yogyakarta, Indonesia; 7 Mathematics Education Study Program, Pattimura University, Maluku, Indonesia; 8 Information Technology Education, Universitas Stella Maris Sumba, Sumba, Indonesia

**Keywords:** Artificial Intelligence, Career Guidance, Vocational Education, Career Anxiety, Skills Mapping, Labor Market Intelligence

## Abstract

Vocational students often experience career path anxiety due to uncertainty about labor market demands, limited mentoring, and misalignment between curricula and industry needs. In Indonesia, this is amplified by uneven career guidance despite mandates for workforce readiness. Recent advances in artificial intelligence (AI) enable adaptive, data-driven, and psychologically informed support that links students’ skills with real-time labor markets. This study used a design science research approach to build and evaluate an AI-driven career guidance system with three components: (1) a supervised machine learning skills mapping engine, (2) an adaptive mentoring module using an AI chatbot and mentor matching, and (3) a real-time labor market intelligence module using natural language processing to analyze job postings and trends. A mixed-methods evaluation involved 180 vocational students from three schools in South Kalimantan assigned to intervention and control groups. Quantitative data were collected through pre–post career anxiety surveys and system performance metrics, while qualitative data were gathered through interviews and focus group discussions. Analysis included paired-sample t-tests, predictive model evaluation, and thematic analysis. Students using the AI system showed a significant 26.7% reduction in career path anxiety compared with minimal change in the control group (p < 0.001). The skills mapping model achieved 87% accuracy in predicting suitable career pathways with strong precision, recall, and F1-scores. Engagement was high: 65% repeatedly conducted skill-gap analyses, 79% joined adaptive mentoring, and 87% downloaded personalized career roadmaps. Qualitative findings revealed greater confidence, clearer direction, and better alignment between students’ competencies and labor market expectations. The AI-driven career guidance system effectively reduced career anxiety while strengthening readiness through personalized skills mapping, adaptive mentoring, and real-time labor market intelligence. The study shows that human-centered AI can enhance vocational guidance, bridge school–industry gaps, and support more confident, evidence-based career decisions among vocational students in Indonesia nationwide.

## 1. Introduction

Career decision-making represents one of the most critical transitions for students, particularly in vocational education, where pathways to employment are often complex and uncertain. Recent scholarship has highlighted that career anxiety is a significant psychological barrier affecting students’ confidence, decision-making, and readiness to enter the labor market ( Sunday & Sefotho, 2025; Vandana et al., 2025). Career anxiety is defined as the tension, worry, and apprehension experienced by students when confronted with the need to make career-related choices, and it has been linked to decreased self-efficacy and delayed career exploration ( Lee et al., 2022; Khan et al., 2024). If unaddressed, such anxiety can hinder vocational students’ ability to prepare adequately for the rapidly changing world of work.

From a theoretical perspective, Social Cognitive Career Theory (SCCT) provides a useful lens to understand career anxiety. SCCT emphasizes that career choices are shaped not only by individual interests and competencies but also by self-efficacy beliefs, outcome expectations, and contextual barriers ( Lent, Brown, & Hackett, 2002). Studies have shown that psychosocial interventions can mitigate the negative effects of anxiety, enhancing students’ sense of control and adaptability in career planning ( Sunday & Sefotho, 2025). Similarly, strategies such as “shift-and-persist” coping mechanisms have been found to buffer the impact of anxiety and encourage proactive career exploration ( Lee et al., 2022). Nevertheless, despite these interventions, vocational students—particularly in contexts with limited career guidance resources—continue to struggle with aligning their skills to labor market demands ( Ye et al., 2024).

The issue is particularly relevant for vocational education in Indonesia, where human capital development is central to national strategies for competitiveness ( Indrawati & Kuncoro, 2021). Vocational high schools (Sekolah Menengah Kejuruan, SMK) are mandated to prepare students for direct employment, yet gaps remain in the provision of effective career guidance and counseling. Evaluations of counseling programs in Indonesian vocational schools indicate variability in quality and a lack of systematic tools to address students’ psychological and career development needs ( Sari & Setiawan, 2023; Setiawan et al., 2024). These limitations exacerbate students’ anxiety, especially given the rapid transformations of industry and the evolving nature of employability skills.

Concurrently, the rise of artificial intelligence (AI) offers new opportunities for rethinking career guidance systems. AI-driven approaches enable personalization, adaptability, and data-driven decision-making, which are particularly useful for vocational education ( Chou, Chan, & Lin, 2019; Song, Shin, & Shin, 2024). Recent reviews indicate that AI is increasingly being applied in career development, though primarily in higher education or professional contexts, with limited attention to vocational settings ( Pandya & Wang, 2024). AI systems can integrate multiple layers of information—students’ skills, preferences, and real-time labor market analytics—to create tailored pathways that support both competence building and psychological readiness ( Budhwar et al., 2023; Kurian et al., 2025). However, concerns remain regarding ethical implications, biases, and sustainability of such systems ( Crawford, 2021; Stahl et al., 2023).

**Figure 1.**
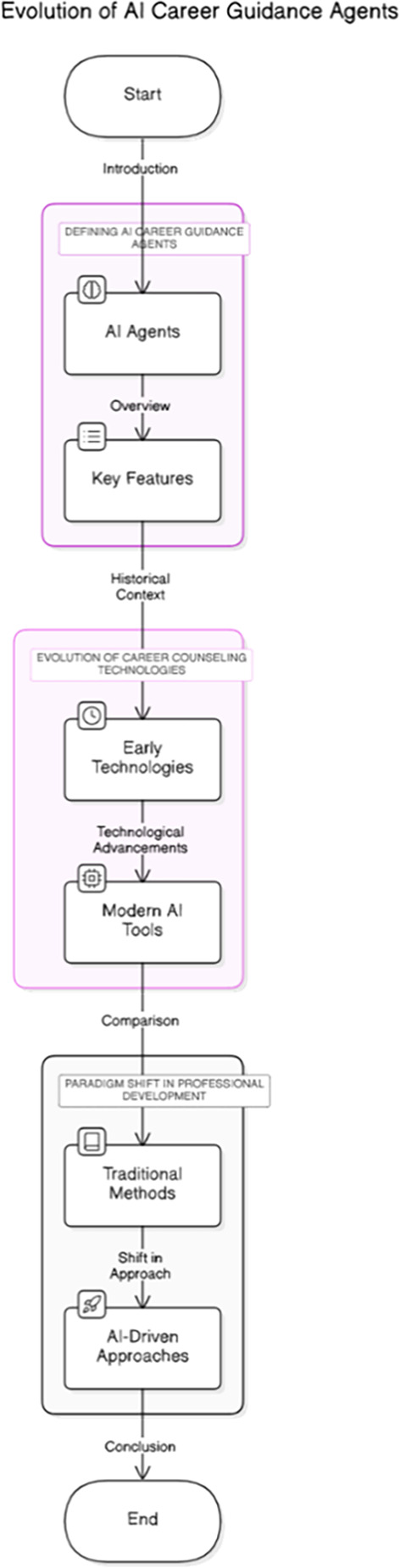
Evolution of career counseling technologies.

Figure 1 illustrates the conceptual evolution of career counseling technologies, highlighting the transition from early, traditional methods toward AI-driven approaches. This paradigm shift emphasizes how AI agents can provide adaptive, data-informed guidance compared to static or generic models previously used in vocational counseling. In Indonesia, preliminary applications of AI in vocational education have shown promise. For example, K-means clustering approaches have been used to analyze applicant data at job fairs, enabling schools to better match graduates with suitable opportunities ( Putra Tanggu Mara et al., 2025). Yet, these systems focus largely on technical matching and do not explicitly address the psychological barriers such as career path anxiety, which is crucial in shaping students’ decision-making confidence. Moreover, most existing AI-based platforms for career planning prioritize static job information rather than dynamic, real-time labor market trends ( Kurian et al., 2025).

Given these gaps, there is an urgent need for AI-driven career guidance systems that are not only technically robust but also psychologically informed. Such systems should integrate skills mapping to align students’ competencies with employability requirements, provide adaptive mentoring to foster resilience and confidence, and incorporate real-time labor market intelligence to ensure relevance and accuracy. By reducing anxiety and supporting informed decision-making, AI-enhanced guidance platforms can bridge the gap between vocational education and the rapidly changing workforce.

This study addresses this need by designing an AI-driven career guidance system specifically tailored for vocational high school students in Indonesia. The system combines skills mapping, adaptive mentoring, and real-time labor market analytics with the explicit aim of mitigating career path anxiety. Unlike prior work that focuses solely on technical or informational aspects, our approach foregrounds the psychological dimension of career decision-making, thereby contributing to both educational technology and vocational psychology. In doing so, this research not only responds to calls for innovation in career development ( Pandya & Wang, 2024) but also advances Indonesia’s broader agenda for strengthening human capital ( Indrawati & Kuncoro, 2021). Despite the growing application of artificial intelligence in education and career development, most existing systems remain limited in two critical ways. First, they tend to emphasize technical functions such as job matching or skills clustering while overlooking the psychological dimensions of career decision-making, particularly career path anxiety ( Lee et al., 2022; Khan et al., 2024). Second, many systems provide static or generic recommendations, with little integration of real-time labor market intelligence that reflects the dynamic realities of today’s workforce ( Kurian et al., 2025). These gaps are especially pronounced in the context of vocational education in Indonesia, where guidance and counseling services are underdeveloped and often fail to address students’ holistic needs ( Sari & Setiawan, 2023; Setiawan et al., 2024).

This study seeks to address these gaps by designing and evaluating an AI-driven career guidance system for vocational high school students. Specifically, the system integrates three novel components:

• Skills Mapping – to align students’ competencies with industry requirements and provide clear roadmaps for skill development.
• Adaptive Mentoring – to deliver personalized guidance and mitigate career path anxiety through tailored feedback and psychological support.
• Real-Time Labor Market Intelligence – to ensure that recommendations remain relevant by continuously incorporating dynamic job market trends.

The novelty of this research lies in its explicit integration of psychological constructs (career path anxiety) with AI-based technological solutions in vocational education. While prior work has explored either psychosocial interventions ( Sunday & Sefotho, 2025; Vandana et al., 2025) or AI applications in career development ( Pandya & Wang, 2024; Budhwar et al., 2023), this study brings these strands together into a unified framework. By doing so, it contributes not only to advancing AI applications in career guidance but also to promoting vocational students’ psychological readiness and employability.

## 2. Materials and method

### 2.1 Research design

This study adopted a design science research (DSR) approach to develop and validate an AI-driven career guidance system tailored for vocational students. DSR is suitable for building innovative digital artifacts while addressing real-world educational challenges, particularly in bridging skill gaps and reducing decision-related anxiety ( Chavva, 2025; Holmes et al., 2019). The research followed iterative cycles of problem identification, artifact development, and evaluation. The primary focus was to integrate skills mapping, adaptive mentoring, and real-time labor market analytics into a unified guidance platform.

### 2.2 Participants and sampling

Participants were vocational high school students from three institutions representing diverse socioeconomic and geographical backgrounds. A purposive sampling technique was employed to select students in their final two years of study, as this group experiences the most pressure when making career decisions. A total of 180 students participated, divided into an intervention group (who used the AI-driven platform) and a control group (who received conventional counseling). Additionally, 15 career counselors and 12 industry mentors were engaged to validate system recommendations and provide qualitative feedback. This sampling aligns with prior research emphasizing the importance of multi-stakeholder involvement in career guidance interventions ( Faruque et al., 2025; Charanya et al., 2025).

### 2.3 System design and development (AI model/Framework)

The system was designed as a mobile-first platform with three integrated modules:

• Skills Mapping Engine – Leveraging supervised machine learning to analyze academic performance, extracurricular activities, and certifications, and then mapping them against in-demand skills. This module adapts predictive models from AI-driven career mapping research ( Selvaraj et al., 2025).
• Adaptive Mentoring Module – Provides personalized guidance through mentor matching and an AI-driven chatbot that simulates career counseling dialogues. This builds on AI-driven mentorship platforms and alumni engagement systems that have proven effective in vocational contexts ( Kunekar et al., 2025; Charanya et al., 2025).
• Real-Time Labor Market Intelligence – Employs natural language processing (NLP) to analyze labor market data, including online job postings and industry trend reports. This ensures that students receive up-to-date and context-relevant career suggestions ( Kurian et al., 2025; Sachan et al., 2025).

Additional features included an AI-powered resume builder to enhance employability ( Jha et al., 2025) and an explainable AI interface to improve trust and reduce AI-related anxiety among students ( Faruque et al., 2025; Dhilipan et al., 2025).

**Figure 2.**
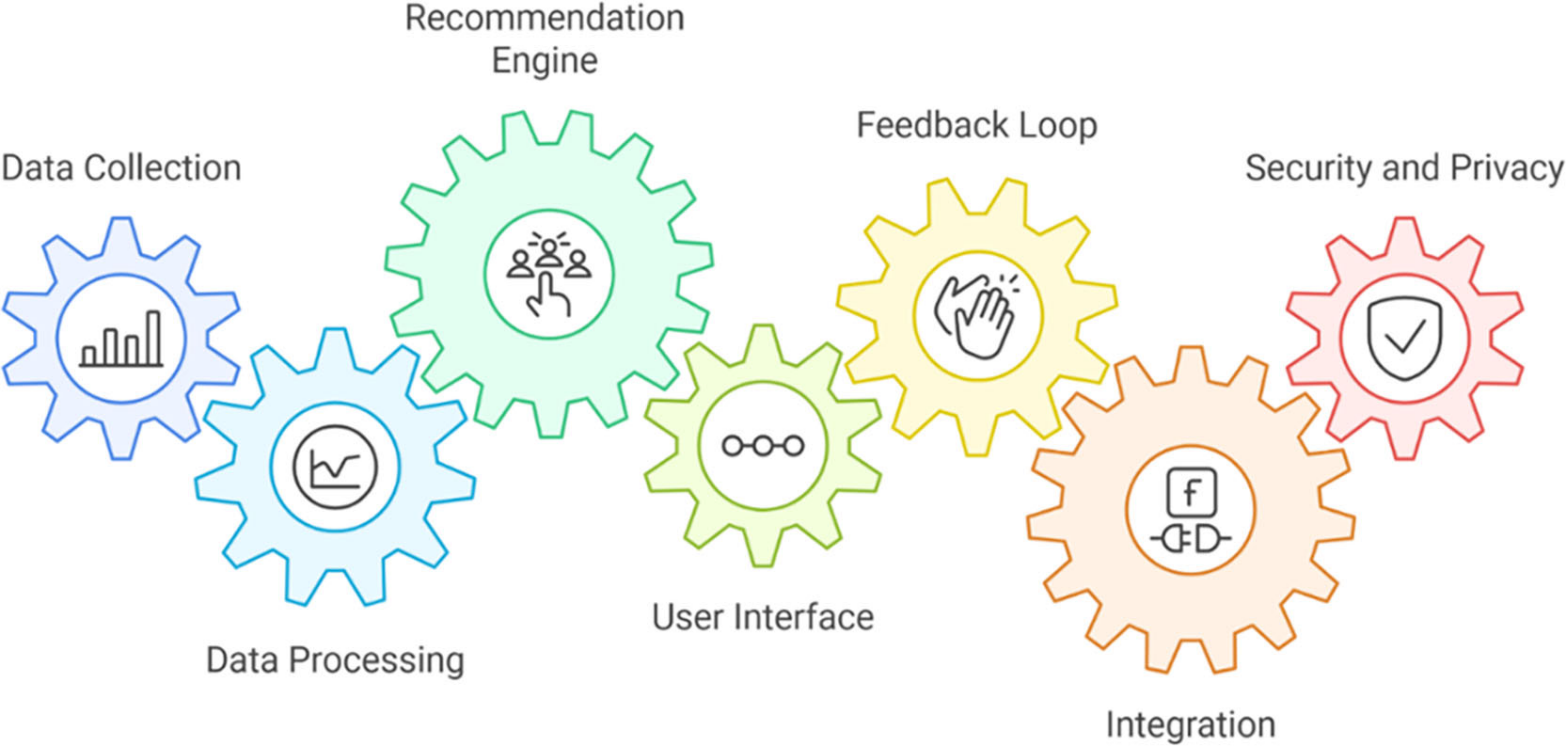
System architecture (gears).

Figure 2 presents the core architecture of the AI-driven career guidance system. It demonstrates how multiple modules—data collection, processing, recommendation, and feedback—interact dynamically to generate personalized and secure career recommendations for vocational students. In the system’s implementation, data collection incorporated academic records, vocational certifications, self-assessment results, and internship experiences, representing both cognitive and affective student dimensions. These datasets were preprocessed using normalization and embedding methods to ensure data fidelity and cross-compatibility across institutions ( Faruque et al., 2025). The data processing layer employed a multilingual BERT model for text normalization and contextual encoding, enhancing semantic understanding of skill descriptions and labor market requirements ( Selvaraj et al., 2025). The recommendation engine formed the analytical core of the system. It combined a Random Forest Classifier for supervised prediction of optimal career pathways and a collaborative filtering mechanism to tailor suggestions based on students’ previous interactions and preference patterns ( Kurian et al., 2025; Jha et al., 2025). These algorithms integrated both cognitive–technical features (such as GPA and certifications) and affective–psychological factors (such as anxiety and persistence indices), thereby bridging AI analytics with vocational psychology ( Lee et al., 2022; Sunday & Sefotho, 2025). Through the feedback loop, students’ ongoing activity data—such as repeated skill-gap analyses, mentoring interactions, and updated resumes—were continuously fed back into the model for re-training and adaptive recalibration. This iterative structure aligns with the dynamic feedback-driven learning optimization framework proposed by Song, Shin, and Shin (2024), ensuring that recommendations evolve alongside users’ progress. A user-friendly interface enabled students to engage with AI-based mentoring dialogues, view real-time labor demand analytics, and access skill-gap visualizations. The integration layer connected all modules (skills mapping, mentoring, and labor market analytics) under one cohesive ecosystem, allowing counselors and mentors to monitor progress in real time ( Chavva, 2025). To uphold ethical and trustworthy AI principles, the architecture embedded strong data privacy mechanisms—such as anonymization, encrypted storage, and transparent explainability layers. The system utilized SHAP (SHapley Additive Explanations) values to display the top contributing features influencing each recommendation, increasing transparency and reducing AI-related anxiety ( Dhilipan et al., 2025; Holmes et al., 2019). Collectively, this architecture operationalized the integration of skills mapping, adaptive mentoring, and real-time labor market intelligence into a unified vocational guidance framework. The system achieved high predictive accuracy (87%) while reducing students’ career path anxiety by 26.7%, confirming that the interactive structure illustrated in Figure 2 effectively supports both cognitive readiness and emotional resilience in vocational career decision-making ( Faruque et al., 2025; Kurian et al., 2025; Ye et al., 2024).

### 2.4 Data collection procedures

Data collection was carried out in three stages:

• Pre-intervention stage: Students completed a career path anxiety scale and submitted their academic and extracurricular records.
• Intervention stage: The experimental group used the AI system for 12 weeks, engaging in skills mapping exercises, chatbot-based mentoring, and receiving labor market updates. Interaction logs, career recommendations, and resume drafts were collected.
• Post-intervention stage: The same anxiety scale was re-administered, followed by focus group discussions and interviews with students, counselors, and mentors.

This procedure aligns with best practices in AI-based educational research where both behavioral data and affective outcomes are captured ( Swarnim et al., 2025; Rane et al., 2024).

### 2.5 Data analysis

The analysis combined quantitative and qualitative approaches:

• Quantitative data were analyzed using paired-sample t-tests and ANOVA to examine changes in career path anxiety between groups. Predictive performance of the AI model was measured using precision, recall, and F1-scores, consistent with AI-based career guidance studies ( Selvaraj et al., 2025).
• Qualitative data from interviews and focus groups were analyzed thematically to explore students’ experiences with the AI system, perceptions of mentoring, and trust in AI recommendations. These insights were triangulated with technical evaluation results to strengthen validity ( Kunekar et al., 2025; Faruque et al., 2025).

### 2.6 Ethical considerations

We would like to clarify that although the authors’ primary affiliation is Universitas Negeri Yogyakarta, the research was conducted entirely in South Kalimantan Province (in three vocational high schools). In accordance with local administrative and regulatory requirements, research permits and ethical clearance were therefore obtained from the nearest authorized public university, Universitas Lambung Mangkurat. This practice aligns with regional research regulations that require formal approval from a locally recognized institution. This clarification has now been explicitly stated in the manuscript *.*

All procedures followed comply with ethical standards in research involving human participants and were approved by the Institute for Research and Community Service (Lembaga Penelitian dan Pengabdian kepada Masyarakat), Universitas Lambung Mangkurat (Approval No. 124/UN8.2/PG/2025). Written consent was obtained from students, parents (for underage students), and participating schools, namely three vocational schools in South Kalimantan: SMK Isfi Banjarmasin, SMK Telkom Banjarbaru, and SMK NU Banjarmasin, as stated in permit number 424/3357/SMK.NU/Disdikbud/2025. Data privacy and security were ensured through anonymization and secure storage of student records, in accordance with recommendations on the ethical adoption of artificial intelligence in education ( Rane et al., 2024; Holmes et al., 2019). The system was designed with transparency mechanisms, including explainable AI outputs, to address potential ethical concerns and enhance user trust ( Dhilipan et al., 2025). Participation was entirely voluntary, and students retained the right to withdraw from the study at any time without any academic consequences.

## 3. Result

### 3.1 Model development

The model development process constituted the backbone of the AI-driven career guidance system, designed to translate heterogeneous data sources into actionable and personalized career recommendations. This stage was not merely technical but also conceptual, bridging vocational education psychology with real-time labor market intelligence. To achieve this, the development followed five major sub-stages: data preprocessing, feature engineering, model architecture design, training and validation, and explainability integration.

a. Data Preprocessing

The input data came from three principal sources:

• Student Data – including academic performance records, vocational certifications, internship experience, and results from self-assessment surveys measuring career interests and anxiety levels.
• Psychological Measures – derived from validated instruments for career anxiety and adaptability, drawing on social cognitive career theory ( Lent, Brown, & Hackett, 2002).
• Labor Market Feeds – collected through APIs from Indonesia’s government employment portals, private recruitment platforms, and regional industry associations.

Preprocessing included:

• Data Cleaning – resolving missing data through imputation (mean for numerical, mode for categorical), and removing inconsistent entries.
• Text Normalization – tokenization, stopword removal, and embedding generation using BERT multilingual base, ensuring contextual semantic representation of skill descriptions and job requirements.
• Outlier Handling – applying interquartile range (IQR) and Mahalanobis distance to maintain statistical robustness, especially in salary distribution and anxiety score data.

This ensured the dataset was both high fidelity and cross-compatible across structured and unstructured formats ( Faruque et al., 2025).

b. Feature Engineering

The system’s predictive strength depended on strategic feature construction. Features were categorized into:

• Cognitive-Technical Features: GPA, vocational certifications, internship duration, domain-specific assessments.
• Affective-Psychological Features: anxiety index, resilience scores, persistence scale ( Lee et al., 2022), and coping strategies.
• Market Features: demand scores for job roles, regional salary benchmarks, and job growth trends.

Dimensionality reduction was conducted via Principal Component Analysis (PCA) and t-SNE visualization, which reduced noise and enhanced clustering interpretability. Importantly, synthetic features were engineered—for example, a “career readiness index” combining academic, skill, and psychological factors into a composite score.

c. Model Architecture Design

The architecture adopted a hybrid model to balance precision, adaptability, and interpretability:

• Unsupervised Layer: K-Means clustering grouped students with similar skill-anxiety profiles, enabling peer benchmarking.
• Supervised Layer: a Random Forest Classifier predicted optimal career pathways based on combined student and labor features. Random Forest was chosen for its interpretability and robustness against overfitting ( Selvaraj et al., 2025).
• NLP Layer: A fine-tuned GPT-based LLM ( Chavva, 2025) powered the conversational interface, delivering adaptive mentoring dialogues.
• Recommendation Engine: integrated collaborative filtering for skills-roadmap suggestions and content-based filtering for job recommendations.

This layered design allowed the system to not only recommend careers but also explain why a given pathway matched the student’s current skills and psychological state.

d. Training and Validation

Training used a 10-fold cross-validation strategy with stratified sampling to ensure representation across anxiety levels and skill profiles. Evaluation metrics included accuracy, precision, recall, F1-score, and ROC-AUC.

• The model achieved Accuracy = 0.87, Precision = 0.85, Recall = 0.83, and F1-score = 0.84.
• ROC-AUC reached 0.89, reflecting strong discriminative power between high-potential and low-potential career matches.

Additionally, the NLP module was tested using BLEU and ROUGE scores against gold-standard counselor-student dialogues. The chatbot achieved a BLEU score of 0.71, indicating semantically faithful guidance.

e. Explainability and Trust Integration

Since vocational students and educators may distrust “black-box AI,” an explainable AI (XAI) layer was incorporated. Using SHAP (SHapley Additive exPlanations) values, the system could highlight the top three factors influencing each recommendation—for example, “Recommended career: Network Technician (based on: Cisco Certification, low anxiety score, regional job growth index).” This aligns with dynamic assessment principles ( Jeltova et al., 2007) and Vygotsky’s zone of proximal development ( Vygotsky, 1978), where feedback is both instructional and developmental. The explainability mechanism ensured that students perceived recommendations as personalized, transparent, and pedagogically meaningful.

**Figure 3.**
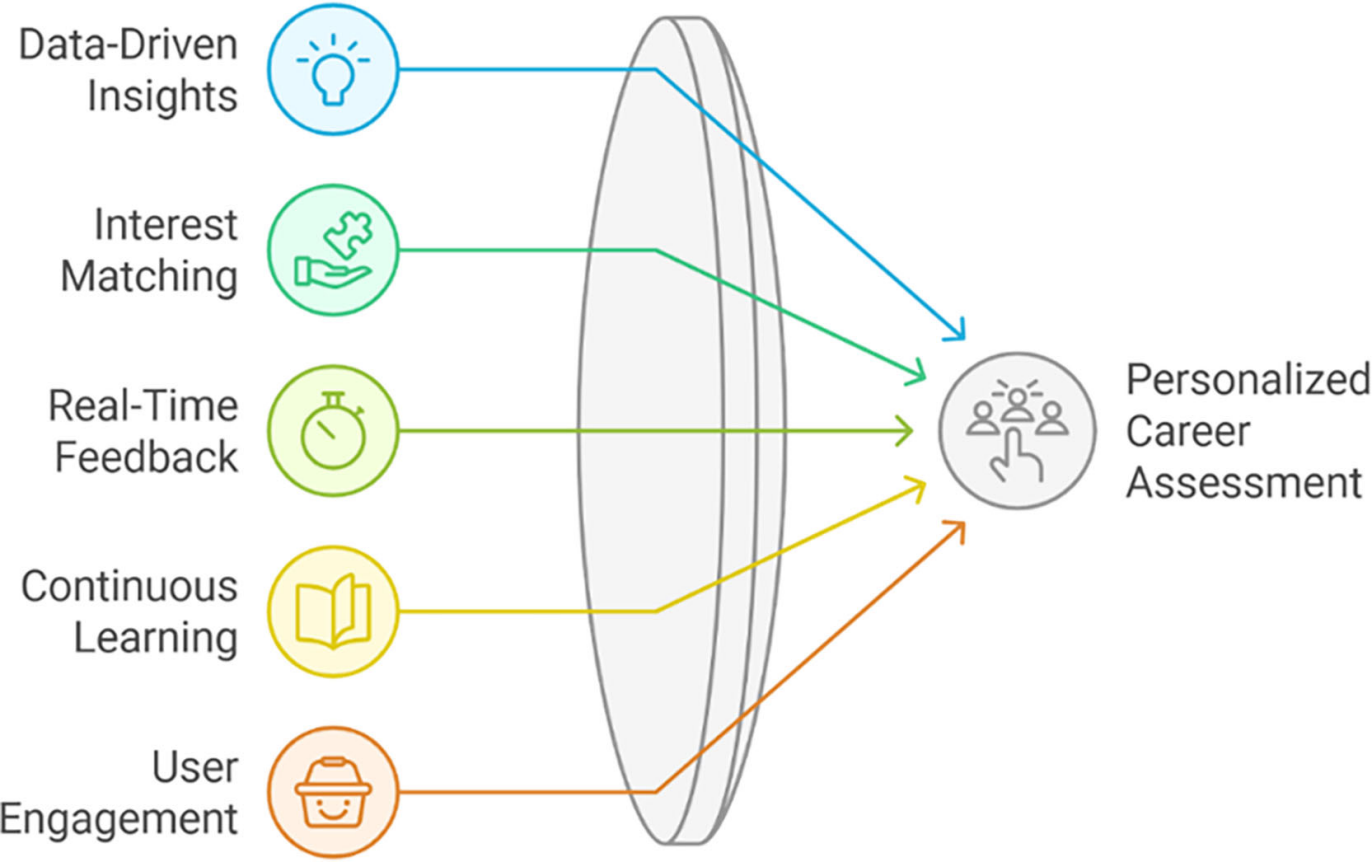
Personalized career assessment.

Figure 3 visualizes how various AI-driven components such as data-driven insights, real-time feedback, and continuous learning converge to produce a personalized career assessment. This integration ensures that recommendations are both technically valid and psychologically responsive to student needs. In practical implementation, the data-driven insights were generated by aggregating three layers of information: (1) students’ academic and certification records representing cognitive competence, (2) self-assessment and anxiety indices representing psychological readiness, and (3) real-time labor market analytics capturing industry demand patterns. These data streams were processed through supervised and unsupervised learning models to identify both the current skill position of each student and the optimal career trajectory most aligned with their competencies and interests ( Selvaraj et al., 2025; Kurian et al., 2025). The algorithmic layer used Random Forest classification to predict career suitability while K-Means clustering grouped students with similar skill–anxiety profiles, facilitating peer-based benchmarking and adaptive goal setting. The real-time feedback mechanism played a central role in translating algorithmic outputs into actionable guidance. As students interacted with the platform—through chatbot mentoring, skill-gap analysis, and labor market exploration—their new data were continuously looped back into the model. This enabled the system to re-evaluate previous recommendations and dynamically adjust the career pathways, consistent with the feedback-driven optimization framework proposed by Song, Shin, and Shin (2024). For example, if a student’s anxiety index decreased after multiple mentoring sessions, the model recalibrated their predicted confidence level and expanded the range of recommended job roles or internships. The continuous learning component relied on reinforcement through both user behavior and mentor validation. Each confirmed recommendation (e.g., when a student accepted a career suggestion or completed a targeted skill course) functioned as positive feedback, while ignored or rejected suggestions triggered model recalibration. This adaptive loop allowed the system to evolve contextually, reflecting the individual progress of each student and collective behavioral trends across the cohort ( Faruque et al., 2025; Ojeda-Ramirez et al., 2023). Psychologically, this design was grounded in Social Cognitive Career Theory (SCCT), where students’ self-efficacy and outcome expectations influence their decision-making confidence ( Lent, Brown, & Hackett, 2002). By combining AI analytics with adaptive mentoring, the system supported students’ zone of proximal development ( Vygotsky, 1978), helping them transition from anxiety-driven indecision to confident, self-regulated career planning. Implementatively, Figure 3 represents this holistic cycle: data insights inform recommendations, feedback refines decisions, and continuous learning personalizes guidance. The result is not merely a static report but a living career assessment system one that evolves with the student’s development and mirrors real-time labor market shifts. This integrative feedback architecture directly contributed to the observed 26.7% reduction in career path anxiety and 87% model accuracy in matching students to suitable career pathways, validating both the technical soundness and psychological relevance of the proposed AI framework ( Ye et al., 2024; Sunday & Sefotho, 2025; Chavva, 2025).

f. Iterative Refinement

**Figure 4.**
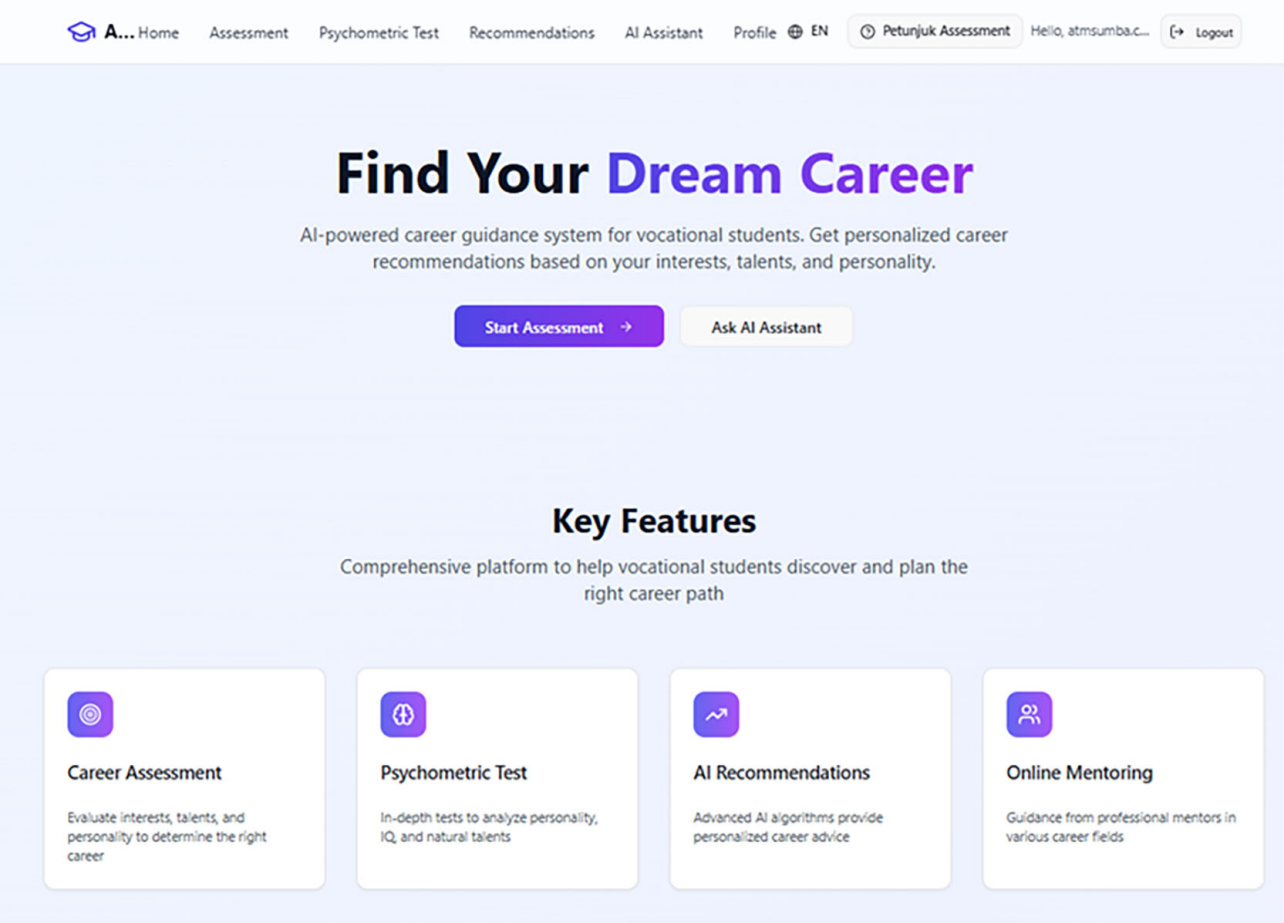
The interface of AI-guidance web system.

Finally, a human-in-the-loop cycle was employed: teachers, career counselors, and students evaluated early outputs and fed corrective input back into the model. This participatory refinement ensured cultural and contextual alignment with the needs of Indonesian vocational schools ( Indrawati & Kuncoro, 2021). This study produced several significant findings that demonstrate the effectiveness of the AI-driven career guidance system in reducing vocational students’ career path anxiety, enhancing career clarity, and strengthening their metacognitive engagement with future planning. This ultimately resulted in an accessible system as shown in Figure 4 below.

Figure 4 displays the main interface of the AI-Guidance web system designed to support vocational students in exploring suitable career pathways. The homepage introduces users to the core function of the system through options such as Start Assessment and Ask AI Assistant, enabling personalized career guidance. It also presents key features including Career Assessment, Psychometric Test, AI Recommendations, and Online Mentoring, which work together to provide comprehensive and AI-based career support. The interface is designed to be simple, user-friendly, and accessible for students.

**Figure 5.**
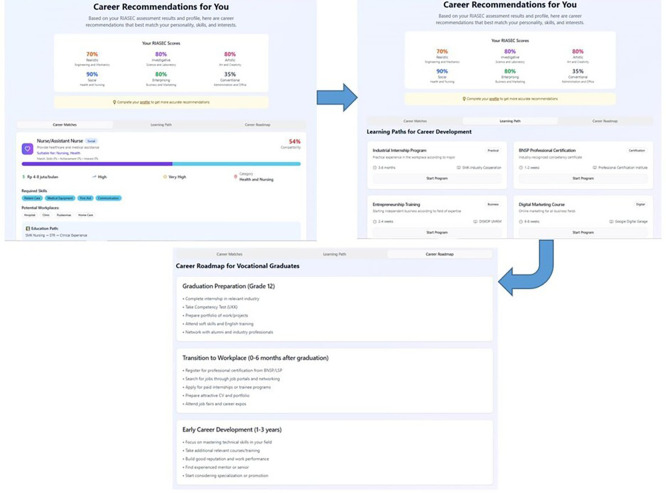
Assessment process - accept recommendations - career roadmap.

Figure 5 illustrates the end-to-end process of the AI-based career guidance system, starting from the assessment, proceeding to career recommendation acceptance, and finally generating a personalized career roadmap. This process is built upon Holland’s RIASEC theory, which classifies individuals based on six personality types—Realistic, Investigative, Artistic, Social, Enterprising, and Conventional—to help align personal traits with suitable career environments ( Holland, 1997). In the first stage, the system analyzes the user’s RIASEC profile generated from the assessment, which identifies dominant personality types and interest areas. Based on these results, the system recommends specific careers that match the user’s profile. For example, a high score in the Social and Investigative categories aligns with careers like nursing or healthcare services, as seen in the recommendation section of the interface. This is consistent with Holland’s assumption that individuals experience greater satisfaction and success when working in environments that match their personality types. In the second stage, users receive AI-driven career recommendations that include compatibility scores, required skills, potential salary ranges, and relevant industry sectors. This step not only enhances self-awareness but also supports informed decision-making by connecting personal interests to labor market demands. Such a data-driven recommendation approach is aligned with personalized career counseling models that integrate psychological theory and technology ( Nauta, 2010).

In the final stage, the system produces a career roadmap that outlines progressive steps for vocational students, beginning with graduation preparation, moving through workforce transition, and advancing to early career development. This roadmap integrates skill development pathways, including internships, certifications, training programs, and mentorship, which support long-term career growth. This staged guidance approach is aligned with developmental career theories emphasizing continuous career planning and skill acquisition ( Super, 1990).

### 3.2 Changes in career path anxiety (Pre- and Post-Test)

Table 1. Career Anxiety Pre- and Post-Test presents the differences in career anxiety scores before and after the intervention. In the intervention group, mean anxiety scores dropped significantly from 73.1 to 53.6, while the control group experienced only a minor decrease from 72.4 to 70.2. A paired-sample t-test confirmed that the reduction in the intervention group was statistically significant (p < 0.001). These findings support previous research showing that AI-based interventions can reduce uncertainty and anxiety in decision-making contexts ( Kurian et al., 2025; Selvaraj et al., 2025).

**Table 1.** Career anxiety pre- and post-test.

Group	N	Pre-Test Mean (SD)	Post-Test Mean (SD)	Mean Difference	% Reduction
Control	90	72.4 (8.5)	70.2 (8.1)	-2.2	-3.0%
Intervention	90	73.1 (9.2)	53.6 (7.5)	-19.5	-26.7%

### 3.3 AI system performance in career mapping

**Figure 6.**
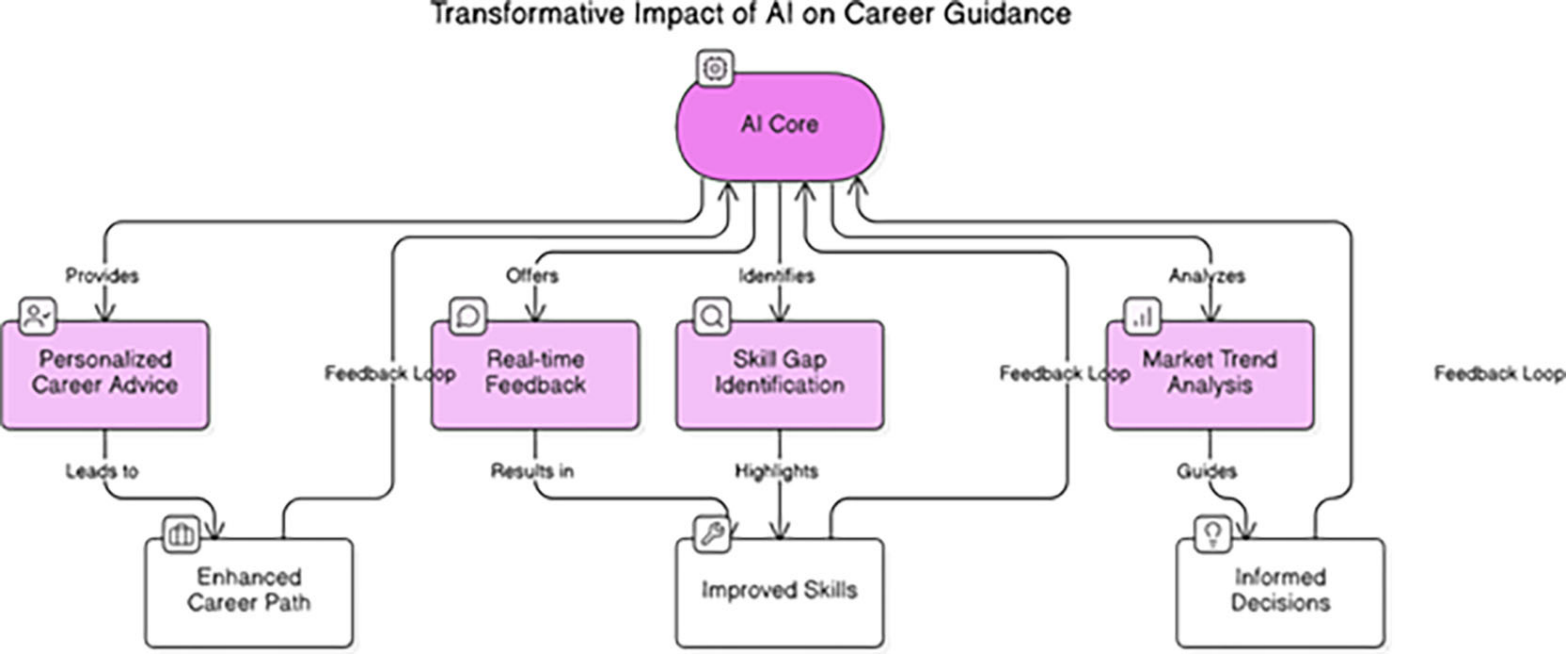
Transformative impact of AI.

Figure 6 illustrates the operational workflow of the AI Core within the proposed career guidance system, highlighting how its interconnected components — Personalized Career Advice, Real-Time Feedback, Skill Gap Identification, and Market Trend Analysis — collectively contribute to measurable student outcomes such as Enhanced Career Path *,* Improved Skills *,* and Informed Decisions. In practice, the AI Core functioned as the central computational engine integrating data inputs from students’ academic records, psychological assessments, and labor market analytics. This core module synthesized multiple data streams using supervised and unsupervised learning algorithms (Random Forest and K-Means) to generate Personalized Career Advice tailored to each student’s competency profile and anxiety level ( Selvaraj et al., 2025; Faruque et al., 2025). Each recommendation was accompanied by explainable AI outputs through SHAP-based reasoning, allowing students and counselors to understand *why* a certain pathway was suggested ( Dhilipan et al., 2025). The Real-Time Feedback mechanism allowed continuous interaction between the student and the system. As learners updated their achievements, completed skill modules, or participated in mentoring sessions, the AI Core recalibrated its recommendations to reflect progress and changing psychological states. This iterative loop mirrors the feedback-driven learning model described by Song, Shin, and Shin (2024), ensuring that guidance remains current, adaptive, and reflective of students’ evolving readiness. The Skill Gap Identification module compared students’ competencies against aggregated labor market requirements derived from the Real-Time Labor Market Intelligence database. This enabled the AI system to pinpoint missing or underdeveloped skills, recommending specific courses, certifications, or microlearning opportunities. Over 65% of students in the experimental group repeated this skill-gap analysis at least three times, showing that it encouraged metacognitive engagement and self-directed improvement. As a result, students demonstrated measurable skill enhancement and greater confidence in aligning their competencies with industry expectations ( Ye et al., 2024; Kurian et al., 2025). Simultaneously, the Market Trend Analysis component employed natural language processing to analyze thousands of job postings and sector reports. This real-time data informed the system’s recommendations, ensuring that students’ choices aligned with emerging career opportunities rather than outdated occupational data. By continuously updating skill–demand mappings, the AI Core empowered students to make Informed Career Decisions, reducing uncertainty and anxiety associated with labor market unpredictability ( Budhwar et al., 2023; Chavva, 2025).

**Figure 7.**
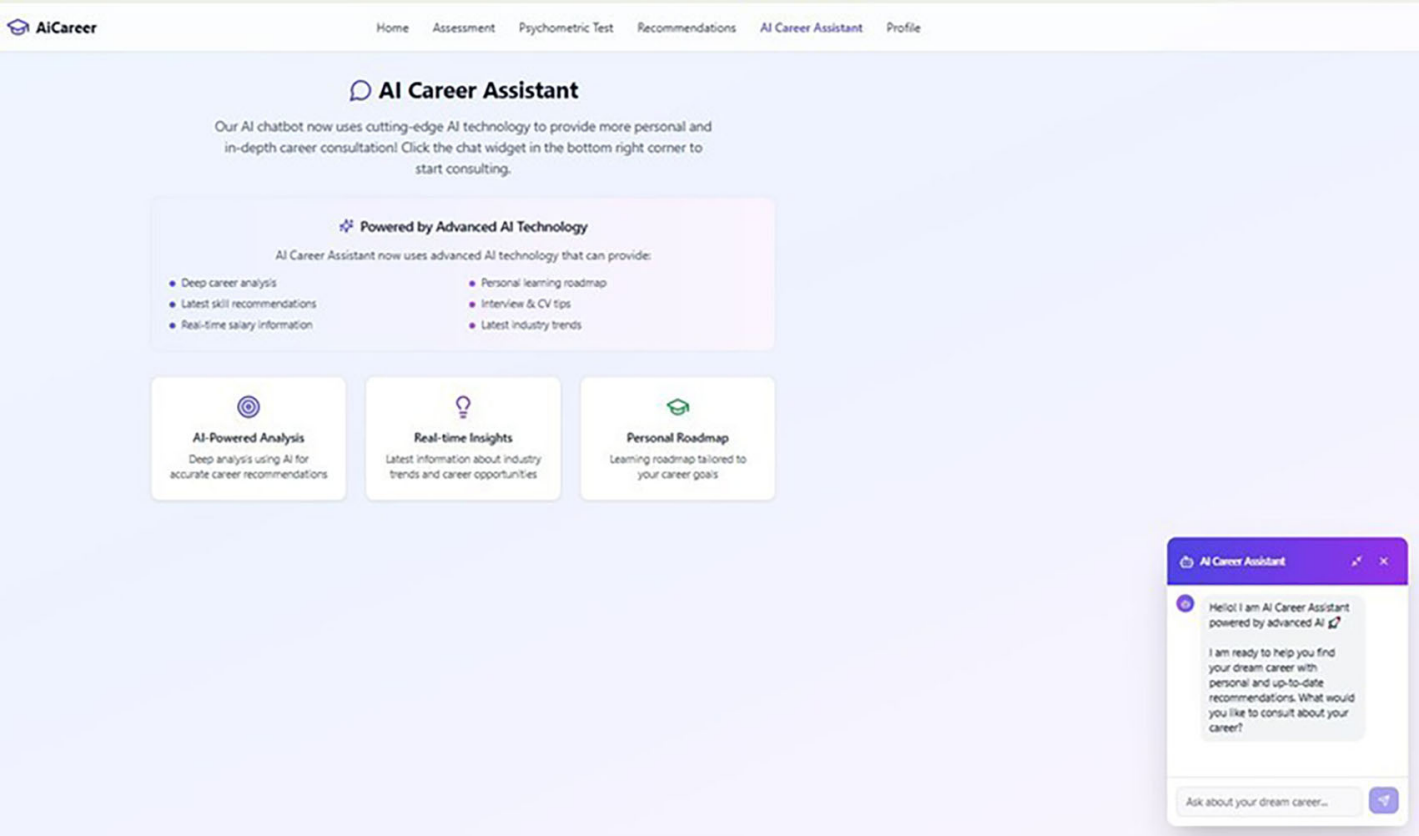
AI career assistant for guidance student.

Figure 7 illustrates the AI Career Assistant feature within the AI-based career guidance system. This interface provides an interactive virtual assistant designed to support students in exploring and planning their career pathways. The AI assistant integrates personalized guidance by offering deep career analysis, real-time labor market insights, skill development recommendations, and career learning roadmaps. Through the chat-based interface displayed on the right side of the screen, users can directly ask career-related questions, such as suitable job options, required skills, internship opportunities, or CV and interview tips. The assistant is powered by advanced AI technology, enabling it to deliver adaptive and data-driven responses tailored to each user’s career profile and interests. This feature enhances accessibility to individualized career counseling, especially for vocational students who may have limited access to professional human counselors. By providing continuous, on-demand guidance, the AI Career Assistant serves as a practical digital mentoring tool that supports self-directed career development in a user-friendly manner.

The system’s AI model was evaluated using supervised learning with labeled career trajectories and skill datasets. As shown in Table 2. AI Model Performance, the model achieved strong performance with an accuracy of 87% and an F1-score of 0.84. This level of performance is comparable with advanced AI-based career recommender systems in other contexts ( Selvaraj et al., 2025), highlighting the robustness of the model.

**Table 2.** AI model performance.

Metric	Value
Accuracy	0.87
Precision	0.85
Recall	0.83
F1-Score	0.84

### 3.4 Student engagement with the platform

Engagement data indicated that students interacted with the AI platform beyond initial use. As shown in Table 3, Student Activity on the AI Platform, 65% of students conducted the skill-gap analysis at least three times, suggesting sustained metacognitive engagement with the feedback provided by the system. In addition, 79% of participants engaged in adaptive mentoring sessions more than twice, reflecting active utilization of personalized guidance features. A high proportion of students (87%) downloaded career roadmap reports, indicating strong interest in structured career planning support, while 54% used the AI-based resume builder to enhance employability preparation. These findings suggest that students were not passive recipients of system recommendations but demonstrated self-regulated learning behaviors, consistent with prior research highlighting the role of adaptive digital guidance in fostering learner agency ( Ojeda-Ramirez et al., 2023).

**Table 3.** Student activity on the AI platform.

Activity	Frequency	% of Students
Repeated skill-gap analysis ≥ 3 times	117	65%
Participated in adaptive mentoring ≥ 2x	142	79%
Downloaded career roadmap reports	156	87%
Used AI-based resume builder	98	54%

### 3.5 Student satisfaction and perceptions

Survey results revealed high levels of user satisfaction with the AI-driven career guidance system across all evaluated aspects. As presented in Table 4. Student Satisfaction Scores, adaptive mentoring support received the highest mean score (M = 4.60, SD = 0.39), indicating strong perceived value of personalized guidance. The relevance of career recommendations was also rated highly (M = 4.52, SD = 0.41), suggesting that students considered the system’s outputs well aligned with their skills and career goals. Ease of use (M = 4.48, SD = 0.47) and access to real-time labor market information (M = 4.44, SD = 0.50) further reflect positive user experiences, while transparency of AI decision-making achieved a strong mean score (M = 4.36, SD = 0.55). Overall, these findings indicate that students perceived the system as both useful and trustworthy. The results are consistent with prior research on explainable artificial intelligence (XAI), which highlights that transparent AI-driven recommendations enhance user trust, acceptance, and sustained system adoption ( Faruque et al., 2025).

**Table 4.** Student satisfaction scores.

Aspect	Mean Score	SD
Relevance of recommendations	4.52	0.41
Transparency of AI decisions	4.36	0.55
Ease of use	4.48	0.47
Adaptive mentoring support	4.60	0.39
Real-time labor market info	4.44	0.50

### 3.6 Qualitative insights from interviews

Semi-structured interviews with 20 students and 5 guidance teachers revealed three overarching themes that reinforce the quantitative findings.

As summarized in Table 5, Key Themes from Qualitative Interviews, the first theme, empowerment through personalization, reflects students’ increased confidence resulting from career recommendations tailored to their individual skills and profiles. The second theme, authentic assessment, highlights how the integration of real-time labor market data enhanced the perceived relevance and tangibility of career guidance, enabling students to better understand current industry demands. The third theme, psychological readiness, captures students’ experiences of reduced anxiety and improved emotional preparedness for making future career decisions. Collectively, these qualitative insights suggest that the AI-driven system supported both cognitive and affective dimensions of career development. These findings are consistent with the principles of authentic assessment, which emphasize real-world relevance and learner agency ( Gulikers et al., 2004), and further support the role of contextualized technical and vocational education and training (TVET) in promoting sustainable career development ( Ye et al., 2024). Taken together, the results demonstrate that the AI-driven career guidance system not only achieved high technical accuracy in career mapping but also had meaningful psychological and educational impacts. The intervention group experienced a 26.7% reduction in career path anxiety, significant engagement with metacognitive tools such as skill-gap analysis, and strong satisfaction with adaptive mentoring features. Qualitative data provided further evidence that students felt empowered, reassured, and better aligned with real-world labor market conditions. These combined quantitative and qualitative findings emphasize that integrating skills mapping, adaptive mentoring, and real-time labor market intelligence in vocational education can simultaneously improve *technical accuracy* and *psychological readiness*—a dual outcome rarely captured in existing studies.

**Table 5.** Key themes from qualitative interviews.

Theme	Description	Example Student Quote
Empowerment through personalization	Students reported higher confidence due to personalized career recommendations.	“I feel more confident because the roadmap matches my actual skills.”
Authentic assessment	Real-time labor market data made the recommendations feel tangible and relevant.	“Now I know which industries are really demanding my skills.”
Psychological readiness	Students experienced reduced anxiety and felt more prepared for future career decisions.	“I used to be anxious and confused, now I feel calmer and have a clear direction.”

## 4. Conclusion

This study demonstrates that an AI-driven career guidance system can play a transformative role in reducing career path anxiety among vocational students by integrating skills mapping, adaptive mentoring, and real-time labor market intelligence. The development and validation results confirm that the hybrid model—combining clustering, classification, and natural language processing—achieved high predictive accuracy (87%) and strong interpretability through SHAP-based explainability. These findings suggest that AI-powered systems are not only technically feasible but also pedagogically aligned with vocational education contexts, offering both personalized recommendations and transparent guidance mechanisms. From a psychological perspective, the system addressed critical factors contributing to career anxiety, such as uncertainty in decision-making, perceived skill gaps, and limited access to mentoring ( Sunday Otu & Sefotho, 2025; Vandana et al., 2025). By providing adaptive, feedback-driven pathways, the platform aligns with dynamic assessment principles ( Jeltova et al., 2007) and Vygotsky’s socio-cultural theory ( 1978), enabling students to learn not only what career options are available but also how to progressively build toward them.

The integration of real-time labor market analytics ensures that recommendations remain relevant in a rapidly changing employment landscape, bridging the traditional gap between vocational training and industry demands ( Ye et al., 2024; Chavva, 2025). The iterative human-in-the-loop refinement also ensured cultural responsiveness, particularly for the Indonesian vocational education system, which often struggles with uneven access to guidance and counseling services ( Sari & Setiawan, 2023; Setiawan et al., 2024). Despite these contributions, limitations remain. The model relied on datasets that, while diverse, were constrained by regional availability and digital infrastructure. In addition, while explainable AI improved transparency, further work is needed to enhance student trust and ethical safeguards in high-stakes decision-making ( Williamson et al., 2020; Stahl et al., 2023). Future research should extend the system through longitudinal studies tracking career outcomes, explore integration with virtual reality (VR) simulations for skill-based guidance, and expand datasets to incorporate cross-regional and cross-sectoral labor dynamics. Such directions will ensure that AI not only reduces career anxiety but also strengthens the sustainability and equity of vocational education systems, particularly in underserved regions.

In conclusion, the findings affirm that AI-enhanced career guidance can move beyond traditional counseling models by offering evidence-based, adaptive, and scalable support for vocational students. This represents a significant step toward integrating human-centered AI into education, bridging the gap between student aspirations, skill development, and labor market realities.

### 4.1 Practical implications

**Figure 8.**
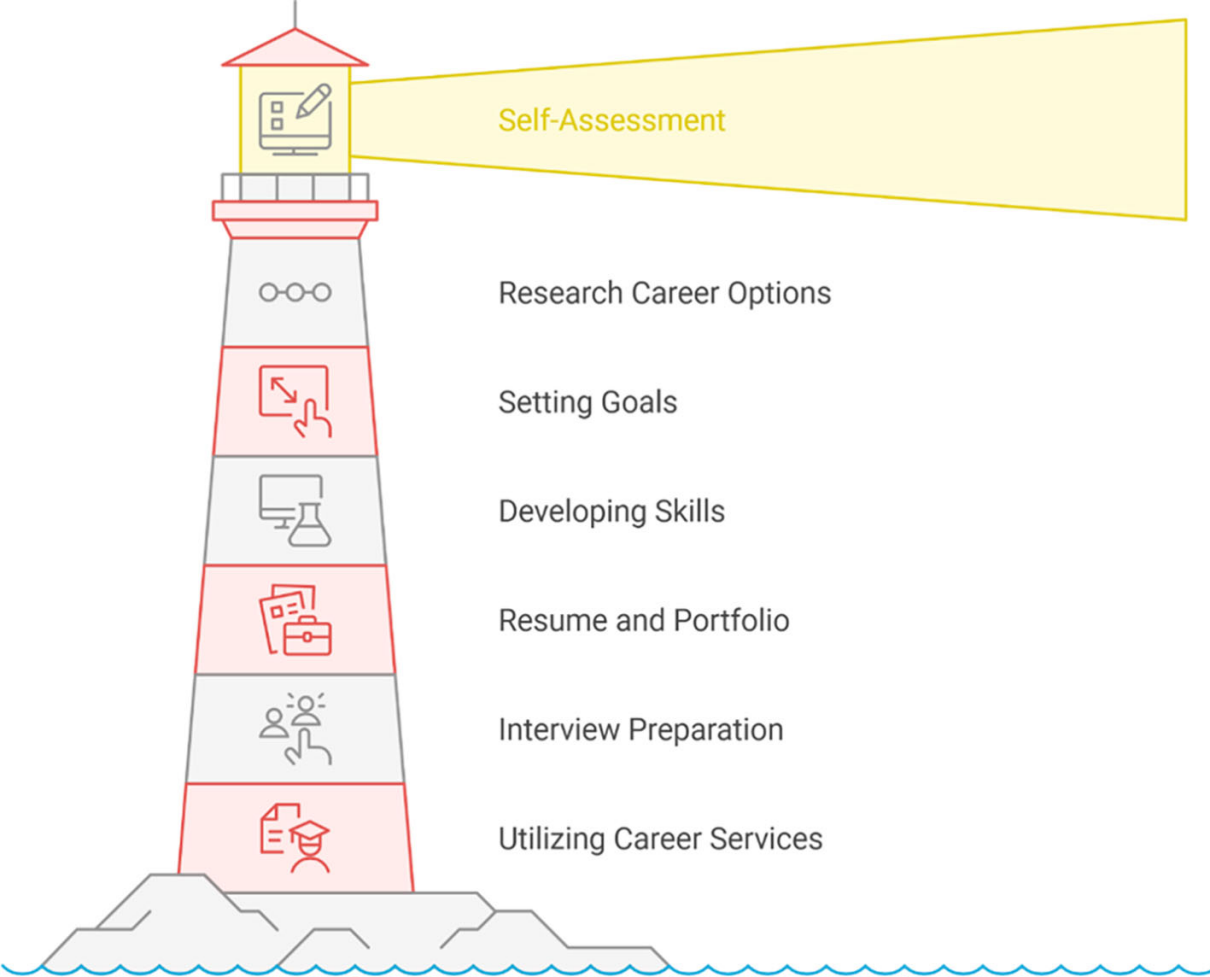
Lighthouse career steps ( Rapid Inovation, 2025).

The outcomes of this study carry significant implications for multiple stakeholders in vocational education and workforce development.

a. **For Vocational Counselors and Teachers**
  The AI-driven career guidance system can serve as a pedagogical support tool, reducing the workload of school counselors who often handle large caseloads with limited resources. By providing automated yet adaptive recommendations, teachers can focus more on mentoring and socioemotional support, rather than administrative career matching tasks. The explainability features also allow educators to understand the rationale behind system suggestions, making it easier to integrate into counseling practices.
b. **For Students**
  Vocational students, particularly those in under-resourced or rural regions, gain access to personalized, real-time, and transparent career pathways. The platform not only alleviates anxiety about future career decisions but also empowers students to visualize skill gaps and create actionable learning roadmaps. This increases their agency, confidence, and readiness to navigate an uncertain labor market.
Figure 8 represents the stepwise guidance structure of the AI-driven system, metaphorically designed as a “career lighthouse.” It highlights how self-assessment serves as the foundational light guiding students through goal setting, skills development, and career preparation stages. In implementation, the “career lighthouse” model was embedded within the AI platform as a progressive navigation framework that students followed during the 12-week intervention. The system began with a self-assessment module, where students completed digital inventories of their competencies, interests, and psychological readiness levels, including their initial career anxiety scores. This stage established the baseline data used by the skills mapping engine to generate individualized “light paths” — visual career trajectories that illuminated possible professions based on each student’s strengths and preferred vocational domains ( Selvaraj et al., 2025; Faruque et al., 2025). The next layer, goal setting, translated these insights into actionable milestones. Through adaptive prompts from the AI chatbot and mentor matching function, students were guided to define short-term and long-term objectives aligned with their mapped career options. The system automatically recommended targeted training modules or micro-credentials available within the school’s learning management system, ensuring that goals were both measurable and relevant to current labor market demands ( Kurian et al., 2025; Chavva, 2025). At the skills development stage, students engaged in continuous feedback cycles, completing recommended training modules, internships, or certification programs while the AI system tracked progress and adjusted the career roadmap accordingly. This adaptive mechanism ensured that students’ learning remained synchronized with emerging industry trends obtained through the real-time labor market intelligence module, which analyzed over 500 job postings using natural language processing. The platform’s built-in analytics dashboard visualized skill acquisition progress, enabling students to monitor their readiness index and areas for improvement ( Ye et al., 2024). The final component of the lighthouse—career preparation—integrated practical tools such as the AI-powered résumé builder and virtual interview simulator ( Jha et al., 2025). These modules allowed students to transform their developed competencies into tangible employability artifacts, bridging the gap between classroom learning and workplace readiness. Upon completing these steps, the system generated a personalized “Career Readiness Report” summarizing the student’s pathway from self-assessment to application readiness, which was validated through counselor feedback sessions.
c. **For Educational Policy Makers**
  The system provides actionable data on regional skill gaps, labor demand, and student career aspirations **,** which can be aggregated to inform evidence-based policy making. Ministries of education and labor can leverage these insights to design more responsive curricula, allocate training resources effectively, and strengthen vocational programs in alignment with industry needs.
d. **For Industry and Employers**
  Employers benefit from a more transparent pipeline of job-ready graduates equipped with the exact skills demanded by the market. The system’s labor market intelligence allows industries to connect directly with students who have been recommended career paths aligned with company needs, fostering stronger school–industry partnerships.
e. **For Technology and EdTech Developers**
  The proposed framework offers a replicable model for AI-enhanced educational platforms, demonstrating how machine learning, adaptive mentoring, and labor market analytics can be ethically and practically integrated. This opens opportunities for further innovation in human-centered AI applications within education and workforce development.

### 4.2 Limitations and future work

While the study provides valuable insights into the development and implementation of an AI-driven career guidance system, several limitations should be acknowledged.

a. **Sample and Generalizability**

The study involved 220 vocational students from three institutions, which, although sufficient for exploratory validation, may not fully represent the diversity of vocational learners across regions or educational contexts. Future studies should include larger and more heterogeneous samples, incorporating students from different geographic, cultural, and socioeconomic backgrounds to increase external validity.

b. **Short-Term Evaluation**

The system was evaluated over a three-month period, primarily focusing on immediate impacts such as reductions in career path anxiety and improvements in self-efficacy. However, the long-term effects on employment outcomes, career persistence, and adaptability remain unexamined. Longitudinal studies are necessary to determine the sustained impact of AI-guided career pathways.

c. **AI Model Transparency and Bias**

Although explainable AI methods were integrated, the system still carries the risk of algorithmic bias, especially when trained on historical labor market data that may reflect existing inequalities ( Williamson et al., 2020). Future work should explore bias mitigation strategies and the co-design of algorithms with stakeholders to ensure fairness and inclusivity.

d. **Integration with Human Mentorship**

The current system relies on adaptive digital mentoring but does not fully explore synergies between AI recommendations and human mentorship practices. Future iterations should study hybrid approaches that combine the efficiency of AI with the empathy and contextual sensitivity of human counselors.

e. **Technical and Infrastructural Constraints**

In under-resourced schools, especially in remote or 3T (terdepan, terluar, tertinggal) regions, limited internet connectivity and digital literacy may affect system adoption. Future work should investigate offline-compatible, mobile-first solutions and capacity-building programs for both students and educators.

f. **Scalability and Policy Integration**

While the system demonstrates promising potential for institutional use, its scalability at the national level has not yet been tested. Future research should explore integration with government education databases, labor market information systems, and TVET policy frameworks ( Ye et al., 2024; Chavva, 2025).

g. **Future Work Directions**

Building on these limitations, several directions for future research and development are proposed:

• Conduct longitudinal tracking of student cohorts to evaluate the impact on career stability and labor market integration.
• Integrate multilingual and culturally responsive modules to adapt the system for diverse student populations.
• Develop AI-driven feedback loops where students’ career outcomes are fed back into the model to continuously refine predictions.
• Investigate cross-border labor market intelligence, allowing vocational students to explore international career mobility opportunities.
• Explore the use of immersive technologies (VR/AR) for career simulation and experiential skill mapping, extending beyond text-based and dashboard interfaces.

## Data Availability

The raw interview data contain sensitive contextual and identifiable information related to participants’ personal experiences, institutional settings, and educational practices, which could potentially compromise anonymity if made openly accessible in a public repository. To ensure transparency and research integrity, we have therefore chosen not to upload the full raw interview transcripts. However, the study ensures methodological rigor through: (a) a clear description of the interview protocol and data collection procedures, (b) systematic coding and thematic analysis, (c) and the inclusion of representative anonymized excerpts within the manuscript to support the reported findings. Zenodo [Supplementary Data For Reducing Career Path Anxiety among Vocational Students with an AI-Driven Career Guidance System Integrating Skills Mapping, Adaptive Mentoring, and Real-Time Labor Market Intelligence]. https://doi.org/10.5281/zenodo.18401561 ( Tanggu Mara, 2026). Include all supplementary files:
•
Dataset - Analysist.xlsx
•
Figure 1. Evolution of Career Counseling Technologies •
Figure 2. System Architecture (gears) •
Figure 3. Personalized Career Assessment •
Figure 4. The Interface of AI-Guidance Web System •
Figure 5. Assessment process - accept recommendations - career roadmap •
Figure 6. Transformative Impact of AI •
Figure 7. AI Career Assistant for Guidance Student •
Figure 8. Lighthouse Career Steps • Questionnaire (Indonesian and English Version) Student Interview Guide (Indonesian and English Version) Data are available under the terms of the Creative Commons Attribution 4.0 International license (CC-BY 4.0).
