# Supplementary material for: AI-Driven Career Guidance to Reduce Vocational Students’ Career Path Anxiety through Skills Mapping, Adaptive Mentoring, and Labor Market Intelligence
Source: F1000Res. 2026 Feb 19;15:298. [Version 1] doi: 10.12688/f1000research.174858.1 (PMC13000396; doi:10.12688/f1000research.174858.1)
Supplement: Peer review history

# Reviewer report: Reviewer response for version 1

**DOI:** 10.5256/f1000research.192796.r463692

**Published:** 2026-03-31

**Authors:** Iwan Kustiawan

**Affiliations:** 1 Universitas Pendidikan Indonesia, Bandung,, West Java, Indonesia

This paper is exceptionally robust, effectively balancing technical rigor with social impact by addressing the "mismatch" between vocational education and industry demands in Indonesia. The use of a Design Science Research approach provides a solid methodological foundation, while the inclusion of specific performance metrics —such as the 26.7% reduction in career anxiety and 87% model accuracy—transforms the paper from a conceptual proposal into a high-impact empirical study. The integration of three distinct AI modules (Machine Learning, Adaptive Chatbots, and NLP-driven Labor Market Intelligence) demonstrates a sophisticated multi-modal technical architecture that is both innovative and practical.

 However, when the methodology mentions 180 participants but the limitations section mentions 220, it creates a significant question regarding data integrity and reporting consistency.

- Is this a reporting error or an attrition issue? Did the study start with 220 students (the "intent-to-treat" population) but only 180 completed the full intervention? If so, the "180" figure should be explained as the final analytical sample, and the reason for the 40 dropouts should be documented.
- Which number is accurate for the statistical power? If the t-tests and p-values were calculated based on 180 students, why does the limitation section claim 220? Conversely, if the system performance metrics used data from 220 users, why does the evaluation section only mention 180?
- Does this impact the "Control vs. Intervention" balance? If 40 students are missing, did they mostly drop out of the intervention group (perhaps due to technical difficulties with the AI) or the control group? A lopsided attrition rate could introduce selection bias, potentially overstating the effectiveness of the AI system.
- Is there a mismatch between quantitative and qualitative samples? Sometimes authors inadvertently combine the total number of survey respondents (e.g., 180) with a separate group of interviewees or pilot testers (e.g., 40), leading to an inconsistent total.

Overall, the Conclusion is academically well-aligned with the standards of high-impact journals. You’ve successfully moved beyond a mere summary by integrating theoretical frameworks (Vygotsky, Dynamic Assessment) and addressing the specific Indonesian context.

 However, to make it truly "journal-ready," there are a few structural and stylistic adjustments needed. While citing literature in the Conclusion is sometimes acceptable to show how your work fits into the broader field, some reviewers prefer the Conclusion to focus exclusively on *your* contributions.

Is the work clearly and accurately presented and does it cite the current literature?

Yes

If applicable, is the statistical analysis and its interpretation appropriate?

Yes

Are all the source data underlying the results available to ensure full reproducibility?

Yes

Is the study design appropriate and is the work technically sound?

Yes

Are the conclusions drawn adequately supported by the results?

Yes

Are sufficient details of methods and analysis provided to allow replication by others?

Partly

Reviewer Expertise:

Electrical Engineering, machine learning, TVET

I confirm that I have read this submission and believe that I have an appropriate level of expertise to confirm that it is of an acceptable scientific standard, however I have significant reservations, as outlined above.

# Reviewer report: Reviewer response for version 1

**DOI:** 10.5256/f1000research.192796.r460908

**Published:** 2026-03-18

**Authors:** Nesrin Menemenci Bahcelerli

**Affiliations:** 1 Near East University, Nicosia, North Cyprus, Turkey

This article discusses an "AI-Powered Career Guidance System" designed to reduce career path anxiety among vocational education (VET) students in Indonesia. The study's key innovation lies in not only offering a technical matching (skill-job) approach but also incorporating a psychological dimension (anxiety management) within the framework of Social Cognitive Career Theory (SCCT). By combining skill mapping, adaptive mentoring, and real-time job market intelligence in a single platform, it fills a gap in the literature regarding "static proposals." The study is well-founded both theoretically (SCCT and Holland's RIASEC theory) and practically. Addressing a specific problem like career anxiety in vocational education using modern AI techniques (NLP, XAI, machine learning) is valuable not only for Indonesia but also for the global field of educational technology.

 While the article details the methods used, further technical details could be added regarding the specific parameters used for the NLP module (GPT-based LLM) and the size of the training dataset. A more in-depth discussion of how potential algorithmic biases in AI models are controlled could be added to the text.

Is the work clearly and accurately presented and does it cite the current literature?

Yes

If applicable, is the statistical analysis and its interpretation appropriate?

Yes

Are all the source data underlying the results available to ensure full reproducibility?

Yes

Is the study design appropriate and is the work technically sound?

Yes

Are the conclusions drawn adequately supported by the results?

Yes

Are sufficient details of methods and analysis provided to allow replication by others?

Yes

Reviewer Expertise:

Management, Education, Tourism

I confirm that I have read this submission and believe that I have an appropriate level of expertise to confirm that it is of an acceptable scientific standard.

# Reviewer report: Reviewer response for version 1

**DOI:** 10.5256/f1000research.192796.r460902

**Published:** 2026-03-12

**Authors:** Maslin Masrom

**Affiliations:** 1 Universiti Teknologi Malaysia, Kuala Lumpur, Malaysia

The article presents an AI-driven career guidance system aimed at reducing career path anxiety among vocational students. The study adopts a Design Science Research approach and evaluates the system with 180 students using both quantitative and qualitative methods. Results indicate a significant reduction in career anxiety (26.7%) and good AI model performance (87% accuracy).

 The article is clearly written, the study design is appropriate, and the conclusions are supported by the results. However, the methodology section should provide more details about the machine learning implementation, dataset structure, and software tools used to improve reproducibility.

 Overall, the study addresses an important topic in vocational education. With minor clarification of methodological details, the manuscript would make a useful contribution to research on AI-based career guidance.

Is the work clearly and accurately presented and does it cite the current literature?

Yes

If applicable, is the statistical analysis and its interpretation appropriate?

Yes

Are all the source data underlying the results available to ensure full reproducibility?

Yes

Is the study design appropriate and is the work technically sound?

Yes

Are the conclusions drawn adequately supported by the results?

Yes

Are sufficient details of methods and analysis provided to allow replication by others?

Partly

Reviewer Expertise:

Data analytics; Data Science

I confirm that I have read this submission and believe that I have an appropriate level of expertise to confirm that it is of an acceptable scientific standard.
